# The influence of imagery vividness on cognitive and perceptual cues in circular auditorily-induced vection

**DOI:** 10.3389/fpsyg.2014.01362

**Published:** 2014-12-03

**Authors:** Aleksander Väljamäe, Sara Sell

**Affiliations:** ^1^Decision, Emotion, and Perception lab, Department of Behavioural Sciences and Learning, Linköping UniversityLinköping, Sweden; ^2^National Center for Rehabilitative Auditory ResearchPortland, OR, USA; ^3^Department of Education, Pacific UniversityForest Grove, OR, USA; ^4^Department of Communication Sciences and Disorders, James Madison UniversityHarrisonburg, VA, USA

**Keywords:** auditorily-induced vection, kinesthetic imagery, visual imagery, spatial sound, illusory self-motion, binaural hearing, circular vection, imagery vividness

## Abstract

In the absence of other congruent multisensory motion cues, sound contribution to illusions of self-motion (vection) is relatively weak and often attributed to purely cognitive, top-down processes. The present study addressed the influence of cognitive and perceptual factors in the experience of circular, yaw auditorily-induced vection (AIV), focusing on participants imagery vividness scores. We used different rotating sound sources (acoustic landmark vs. movable types) and their filtered versions that provided different binaural cues (interaural time or level differences, ITD vs. ILD) when delivering via loudspeaker array. The significant differences in circular vection intensity showed that (1) AIV was stronger for rotating sound fields containing auditory landmarks as compared to movable sound objects; (2) ITD based acoustic cues were more instrumental than ILD based ones for horizontal AIV; and (3) individual differences in imagery vividness significantly influenced the effects of contextual and perceptual cues. While participants with high scores of kinesthetic and visual imagery were helped by vection “rich” cues, i.e., acoustic landmarks and ITD cues, the participants from the low-vivid imagery group did not benefit from these cues automatically. Only when specifically asked to use their imagination intentionally did these external cues start influencing vection sensation in a similar way to high-vivid imagers. These findings are in line with the recent fMRI work which suggested that high-vivid imagers employ automatic, almost unconscious mechanisms in imagery generation, while low-vivid imagers rely on more schematic and conscious framework. Consequently, our results provide an additional insight into the interaction between perceptual and contextual cues when experiencing purely auditorily or multisensory induced vection.

## 1. Introduction

Imagine yourself sitting on a plane, gazing at a beautiful cloudscape and noticing that the engine noise you found so disturbing just a minute ago has suddenly stopped. While such unpleasant experiences occur very rarely, this example highlights the strong association between sound and motion. Sound not only conveys the dynamics of the surrounding environment, but also provides information about its spatial attributes. Hence, systematic research on auditory cues influencing the perception of self-motion and vection (illusory self-motion) is relevant to many areas including postural balance, navigation, and orientation in space, auditory localization, virtual reality and, in particular, simulation of self-motion.

While a large body of research has addressed visually induced vection [e.g., a recent review by Riecke and Schulte-Pelkum (2013)], other sensory cues contributing to this illusion have been studied less intensively. Unfortunately, due to the illusion weakness, and methodological difficulties, such as its vulnerability to conflicting non-auditory cues and/or contextual influences, the research on auditorily induced vection (AIV) still remain scarce and fragmented (Väljamäe, 2009). Similarly to visually induced vection, the sensation of AIV can appear when perceiving the motion of sound objects or fields relative to one's point of audition, e.g., rotating raindrops. However, AIV has been often attributed to purely cognitive, top-down processes, and the weight of contextual vs. perceptual factors in this auditory illusion remains unclear.

The present study addresses the influence of contextual and perceptual factors in the experience of circular auditorily-induced vection. As our *first hypothesis*, we wanted to replicate the results from our previous studies by Väljamäe et al. (2009) and Larsson et al. (2004) showing that the presentation of rotating auditory landmarks such as “a church bell” or “a fountain” sounds are more instrumental in inducing AIV when compared to rotating sound objects recognized as moveable, such as “footsteps” or a “driving bus.” These studies used virtual rotating acoustic 3D soundscapes delivered via headphones, which sometimes leads to perceptual artifacts such as in-head localization or front-back confusion (Blauert, 1997). The present study used the same subset of sounds but rendered now via loudspeaker array. This setup allowed for comparison to past results while eliminating the issue of previously observed artifacts.

Our *second hypothesis* addressed purely perceptual issues of AIV related to spatial hearing mechanisms. Previous studies show that three main cues for discrimination of auditory motion are intensity, binaural cues, and the Doppler effect (Lutfi and Wang, 1999). Intensity cues arise from the changes in sound pressure level emitted by a moving sound source or, alternatively, a moving listener, and are very effective when simulating linear, translational self-motion (Väljamäe et al., 2005). In the case of horizontal (yaw axis) rotational self-motion, the primary spatial cues are binaural which reflect the interaural time and level difference (ITD and ILD) at a listener's ears. While ITD-based cues dominate spatial sound localization below approximately 1600 Hz, ILD-based cues are dominant for higher frequencies (Blauert, 1997). However, it is important to note that despite this frequency dependence, these two binaural cues do not represent two distinct and non-overlapping spatial hearing mechanisms (Grothe et al., 2010).

Several studies have investigated the binaural cues processing while experiencing real or imaginary self-motion. Thurlow and Kerr (1970) found consistent sound localization displacements and nystagmoid eye-movements during visual rotation. Shifts in subjective auditory median plane during experienced illusory and real body self-motion were also reported by Cullen et al. (1992). Similar results were later reported by Lewald et al. (2004), who used passive whole-body rotation. More recently, Otake et al. (2006) studied the performance in ITD and ILD discrimination tests while listeners viewed rotating optokinetic patterns. Interestingly, only ITDs discrimination showed shifts during rotating visual stimuli and these shifts were correlated with nystagmoid eye movements. To the best of the authors knowledge, only one research group directly addressed the contribution of ILD and ITD cues in auditorily-induced body sway (Iwaki et al., 2013). These authors presented sound sources that moved left/right in front of the listener simulating translational self-motion. Unfortunately, this study was inconclusive in terms of specific contribution of these two binaural cues, perhaps, due to the simulated self-motion type and stimulation weakness. In our study, we directly assessed the contribution of ILD and ITD by filtering ecological sounds inducing vection with the hypothesis that low-pass filtered stimuli (ITD cues) will be more instrumental for AIV as compared to high-pass filtered stimuli (ILD cues).

Our *third hypothesis* addressed the influence of mental imagery abilities of the listeners on AIV experience. Our previous research on linear AIV showed that participants' kinesthetic but not visual or auditory imagery scores were significantly interacting with the external cues provided by the moving scene (Väljamäe et al., 2008a), Experiment (1). More specifically, listeners with low-vivid kinesthetic imagery significantly benefited from the addition of metaphorical, non-spatialized sound representing a vehicle engine, elevating their vection experience to the level of high-vivid KI listeners. The effect of such sonic avatar metaphor as an engine sound falls into the category of so-called “implied motion” cues, that also can induce vection and demonstrate clear top-down cognitive influences in the perception of self-motion (see Seno et al., 2012 and references therein). Other cognitive factors, like a prior knowledge of possibility for self-motion provided by instructions or by an experimental setup (Dodge, 1923; Lepecq et al., 1995) or contextual information (Ogawa and Seno, 2014), also facilitate stimuli-induced vection (see Riecke and Schulte-Pelkum, 2013 for a recent review on top-down influences in self-motion simulation). Therefore, in our experiment we included some conditions of imagined self-rotation with or without external auditory cues. Our hypothesis here was that imagery vividness ability of participants would affect the influence of different types of sound stimuli (hypotheses 1 and 2).

## 2. Materials and methods

### 2.1. Participants

Twenty two participants (10 females) with a mean age of 21.1 (*SD* = 2.8 years) took part in the experiment. All participants gave a written consent to participate in the study, as approved by the Aberdeen Proving Grounds Institutional Review Board and the James Madison University Institutional Review Board. Exclusionary criterion included the presence of a hearing loss, a report of abnormal otologic history, and/or balance difficulties. To ensure normal hearing status, all participants were pre-screened via a typical audiometric test battery. These tests include otoscopy, pure-tone audiograms, speech reception thresholds (SRTs), word recognition scores (WRS), and tympanometry in both ears.

### 2.2. Apparatus

The moving sound assessment was performed in the Dome Room of the Environment for Auditory Research (EAR) at Aberdeen Proving Grounds United States Arms facility in Maryland. Participants were taken into the lab and placed in the rotatable chair to increase the plausibility of moving during the sessions. However, the chair remained still during the entirety of the testing. Participants were told to rest their feet on the bar at the bottom of the chair and to keep their head and eyes fixed forward for the duration of the testing; head position was monitored throughout data collection. Having the subjects feet on the bar was to ensure that there was no conflicting somatosensory information during the session. During each experimental condition, subjects wore videonystagmography goggles that blocked all visual stimuli (recorded nystagmus data have not been used in this paper). The participants head was in the center of a horizontal loudspeaker array with a 6 meter diameter. 8 loudspeakers were used from the array that were 45 degrees apart while the other 82 were gated off throughout the testing. An amplitude pan was created between the real loudspeakers to create the simulated auditory motion.

### 2.3. Stimuli

In this study we used 6 sound stimuli that moved circularly around the participant in the clockwise or counterclockwise direction. Each acoustic stimulus was rotated in a full circle at the angular velocity of 60^0^/s around the participant at the average of 75 dB SPL (measured when presented in front) for 75 s. Each waveform envelope had a linear rise and fall time of 750 ms. In this way, the sound appeared to move around the Dome Room circular array at one revolution every 6 s, or 60^0^/s, thus completing approximately 13 full rotations for each stimulus.

The first stimulus, *“click train,”* was a 100 μs click train with a rate of 7 clicks per second making it a sound covering the whole frequency spectrum. The second stimulus, *“footsteps,”* represented a movable sound object and the third stimulus, a *“bus on idle,”* represented an auditory landmark. Both of these stimuli have been used previously in Väljamäe et al. (2009) and Larsson et al. (2004), where they were clearly identified by listeners as movable or stationary sound objects. The other three stimuli were created using a *“bus on idle”* sound: *“a bus on idle, scrambled”* **(BusSC)** had the same overall frequency characteristics but phase scrambling applied to make it unrecognizable, *“a bus on idle, low-pass”* and *“a bus on idle, high-pass”* versions were low-pass and high-pass filtered with a cut-off frequency of 1600 Hz to address ITD and ILD binaural cues impact.

### 2.4. Measures

Illusory self-motion can be assessed reliably using a simple verbal measure, where participants have to rate their subjective sensation of vection strength on a 0 (no self-motion) to 100 (clear perception of physical self-motion) scale (Hettinger, 2002). For example, Kennedy et al. (1996) has shown that participants had no difficulty in translating their “felt” illusory motion into more complex estimations of felt rotation velocity. We used vection intensity as a verbal measure of the illusion. Vection intensity corresponded to the level of the subjective sensation when experiencing self-motion and was measured on a scale from 0 to 100 (0 is the weakest response and 100 is the strongest). In addition, we asked participants to rate the level of sound object's perceived motion state. The still vs. moving response was measured on a rating scale from −5 to +5 (−5 indicating a perceived stationary sound object and +5 indicating a perceived moving sound object), with 0 indicating a point of uncertainty.

Individual data was collected using a shortened form of Betts Questionnaire upon Mental Imagery (BQMI) by Sheehan (1967) in order to investigate the possible influence of the participants' imagination on AIV. The BQMI assesses mental imagery vividness for visual, auditory, kinesthetic, cutaneous, olfactory, and organic senses. For each modality, the participant is asked to generate five mental images and to evaluate their vividness on a 0.7 reverse scale (e.g., maximally vivid, as during the actual task, sensation of *“drawing a circle on paper”* will be given the rating 0). These scores are averaged to form one vividness index for each sense. We collected data only for visual, auditory, and kinesthetic motor imagery.

### 2.5. Procedure and experimental design

The duration of the experiment was around 40 min. Prior to entering the lab, participants were provided with written instructions and were verbally told that they would experience several conditions in which they would hear a sound and may sense that their body was moving. They were also given an opportunity to answer questions, and each acknowledged they understood the instructions. Participants were given a two-way radio communication device and asked to respond with two numbers following each condition; vection intensity and sound object's perceived motion. No prior training or visually-induced vection experience were provided.

The experiment consisted of two parts. In the first part 12 conditions (6 sounds × 2 directions) were presented in a pseudo-random order (Latin square). Here, each of the six sounds were presented twice, in the clockwise and counterclockwise direction. The second, “imagery” part of the experiment, had four conditions (sound on/off × 2 directions) also presented in a pseudo-random order: having the subject imagine that her whole body is rotating clockwise and counterclockwise with or without the accompanying *“bus on idle”* sound. The participants were debriefed and thanked for their participation in the experiment.

### 2.6. Data analysis

All data satisfied the normality criterion as verified using the Kolmogorov-Smirnov test. Analysis of clockwise and counterclockwise direction data showed no significant differences between the rotation direction. Therefore, this data was averaged across these two repetitions.

To test our first two hypotheses, we applied two separate One-Way MANOVAs using ratings on vection intensity and sound object motion state. The use of MANOVA was not related to sphericity violations but aimed to study the effects from combined dependent variables. For these multivariate analyses Wilks′ Lambda Λ was used as the multivariate criterion. Greenhouse-Geisser correction was used to correct for unequal variances, if occurred (Mauchly's sphericity test). Alpha level was fixed at 0.05 for all statistical tests. Bonferroni correction was used for multiple *post-hoc* comparisons. For the third hypothesis, we used One-Way ANOVA for AIV ratings.

For the analysis of the imagery effects, we used a median split to create high and low-vivid imagery participant groups for each of three sensory modalities. These groupings were used as an additional between-subjects factor for the subsequent analyses. For visual imagery (VI) the median split was at 2.7 (scores ranging from 1 to 4), for auditory imagery (AI) − 2.6 (scores ranging from 1.2 to 5.4), for kinesthetic imagery (KI) − 2.8 (scores ranging from 1 to 3.8). The percentage of the same participants in high-vivid imagers groups for all three modalities was 55% (6 out of 11). Comparing groups, the percentage of same high-vivid imagers was 64% (7 out of 11) for AI and VI groups, 64% (7 out of 11) for KI and VI groups, and 73% (8 out of 11) for AI and KI groups.

## 3. Results

Due to the weakness of auditorily-induced vection, in many experimental conditions some participants do not report any self-motion illusion (Väljamäe, 2009). For the first part of the experiment AIV was reported by 55–86% of participants depending on the stimuli type (12–19 participants out of 22), which is comparable with our previous studies on circular AIV [50–75% in Väljamäe et al. (2009)]. 3 participants did not report vection for any of the 12 sound presentations (AIV intensity 0). In the second part of the experiment with imagined self-rotation instructions, all participants reported AIV experience of at least some level of intensity, with 73–95% of participants depending on the experimental condition (16–21 participants out of 22).

### 3.1. Effects of rotating sounds type

In accordance to our first hypothesis, the type of rotating sound had a significant influence on vection intensity ratings. The four stimuli analyzed with One-Way MANOVA were two artificial sounds, *“click train”* and *“a bus on idle, scrambled,”* one movable sound type, *“footsteps,”* and one auditory landmark type, a *“bus on idle.”* The overall effect of stimuli type was significant at *F*_(6, 124)_ = 13.20, *p* < 0.001, Λ = 0.372, ${\hat{\eta }}_{P}^{2}$ = 0.39.

**Figure 1:**
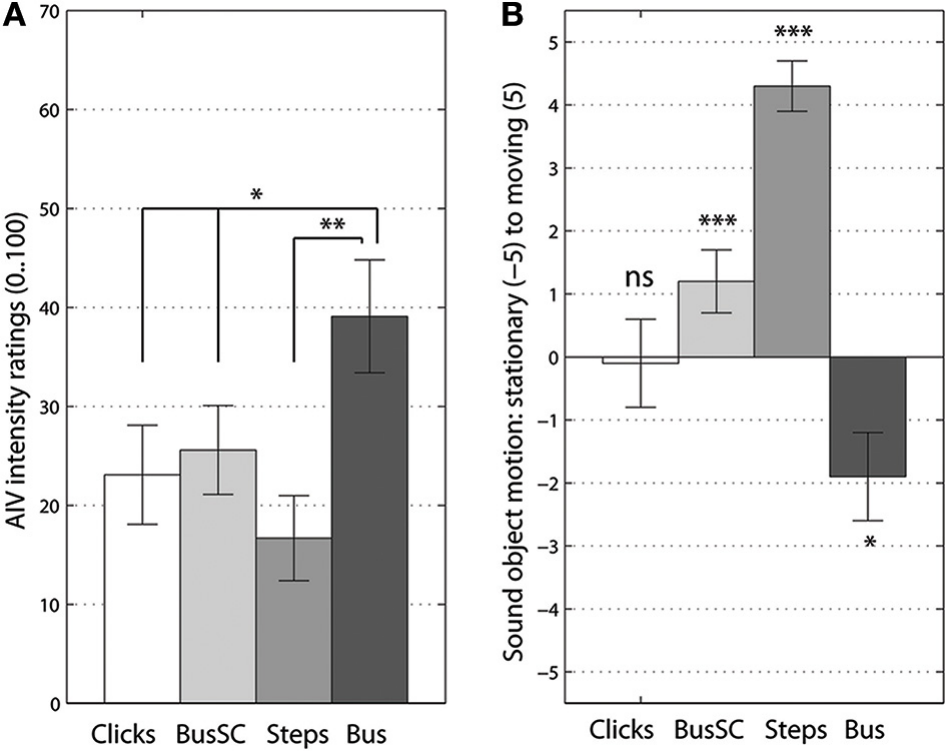
**Vection intensity (A) and perceived sound object's motion ratings (B)**. Significant differences from Bonferroni-corrected pairwise comparisons **(A)** and *t*-tests to 0 **(B)** are marked at *p* < 0.05 (^*^), *p* < 0.01 (^**^) and *p* < 0.005 (^***^) levels. Error bars represent standard error values.

For the AIV intensity ratings the univariate effect was at [*F*_(2, 41)_ = 8.48, *p* < 0.001, ${\hat{\eta }}_{P}^{2}$ = 0.29]. The rotating *“bus on idle”* sound ratings confirmed that auditory landmark type sounds are most instrumental for inducing AIV, see Figure 1A. On the contrary, the movable sound object had the least vection inducing power. *Post-hoc* Bonferroni corrected comparisons showed the significant difference between a *“bus on idle”* and the *“footsteps”* sound at *p* < 0.01. The difference between the bus sound and both artificial sounds was also significant at *p* < 0.05.

The univariate effect of sounds type was also significant for the ratings on perceived sound object's motion [*F*_(3, 55)_ = 28.72, *p* < 0.001, ${\hat{\eta }}_{P}^{2}$ = 0.58]. Both ecological sounds were well-identified as stationary or moving, the scrambled bus sound was judged as moving type and only click train made listeners uncertain of its movability nature. The *“click train”* sound was non-significant when comparing these ratings to 0 (*t* < 1), see Figure 1B. *Post-hoc* Bonferroni corrected comparisons showed significant differences between all four types of rotating sounds. The only non-significant comparison pair, *p* = 0.08, was the difference between the *“bus on idle”* and the *“click train.”*

### 3.2. Contribution of binaural cues

**Figure 2:**
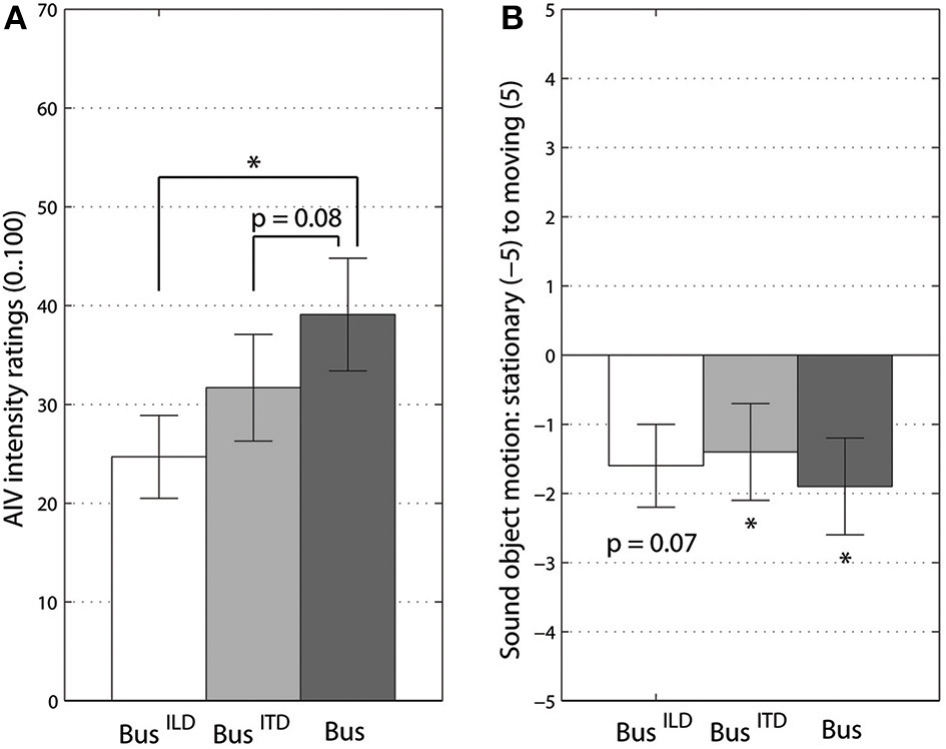
**Vection intensity (A) and sound object's perceived motion ratings (B). Bus^ILD^** stands for high-pass filtered version, **Bus^ITD^** - for low-pass filtered version, **Bus** - for original of *“bus on idle”* sound. Significant differences from Bonferroni-corrected pairwise comparisons **(A)** and *t*-tests to 0 **(B)** are marked at *p* < 0.05 (^*^) level. Error bars represent standard error values.

Supporting our second hypothesis, the frequency content of a rotating sound object had a significant influence on participants' vection intensity ratings. We compared the three frequency content conditions, the original *“bus on idle,”* the low-frequency version with ITD localization cues, *“bus on idle, low-pass,”* and the high-frequency version for ILD localization cues, *“bus on idle, high-pass.”* The overall effect of frequency content in this One-Way MANOVA was significant at [*F*_(4, 82)_ = 3.92, *p* < 0.006, Λ = 0.705, ${\hat{\eta }}_{P}^{2}$ = 0.16]. This effect was only due to AIV intensity ratings [*F*_(1, 34)_ = 6.93, *p* < 0.005, ${\hat{\eta }}_{P}^{2}$ = 0.25] as can be also seen in Figure 2. *Post-hoc* Bonferroni corrected comparisons showed the significant difference between the *“bus on idle, high-pass”* and the original version of a *“bus on idle,”* see Figure 2A. The low-frequency version of the bus sound also had lower AIV intensity ratings, with this difference being close to significance, *p* = 0.08.

Remarkably, all three versions of the *“bus on idle”* sound were judged as coming from a stationary sound object, Figure 2B. There were no significant differences between three bus sound versions for the perceived sound object's motion ratings. All three sounds were also significantly different from 0 level (*t* > 1). This result shows that frequency manipulation did not change the subjective impression and identification of three versions of rotating bus sounds.

### 3.3. Individual differences due to imagery vividness

**Figure 3:**
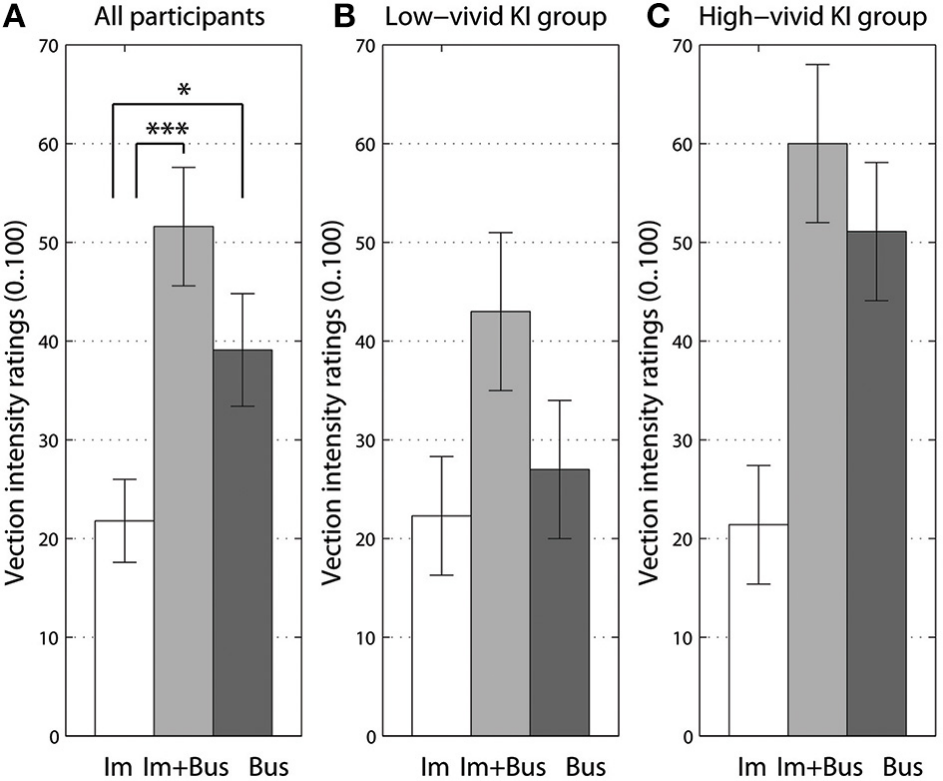
**The effect of imagined body rotation with or without auditory stimuli (A), and data split for low-vivid (B) and high-vivid (C) kinesthetic imagers (KI). Im** stands for imagery of self-rotation, **Bus** - for original *“bus on idle”* sound, and **Im + Bus** for imagery combined with the rotating bus sound. Significant differences from Bonferroni-corrected pairwise comparisons are marked at *p* < 0.05 (^*^), and at *p* < 0.005 (^***^) levels. Error bars represent standard error values.

For the analysis of the effects of imaginary body rotation on vection intensity, we compared three experimental conditions: the *“bus on idle”* and the two conditions where participants either imagined self-rotation in silence or while listening to the rotating bus sound. The two last conditions were tested at the end of the experiment, in a separate block. Since the imagery conditions always implied self-rotation, participants were not asked to rate the sound object's perceived motion. The imagery manipulation was significant [*F*_(2, 39)_ = 14.4, *p* < 0.001, ${\hat{\eta }}_{P}^{2}$ = 0.41]. *Post-hoc* Bonferroni corrected comparisons showed the significant difference between imagery rotation alone and when listening to the *“bus on idle”* sound (*p* < 0.05), and between imagery rotation alone and when combined with rotating bus sound (*p* < 0.001). The data is shown in Figure 3A.

When adding imagery vividness group (high-vivid vs. low-vivid) as an additional between-subjects factor to imagery rotation ANOVA, only the kinesthetic imagery group factor showed a small interaction close to significant [*F*_(2, 36)_ = 2.9, *p* = 0.072, ${\hat{\eta }}_{P}^{2}$ = 0.13]. This interaction can be seen in Figures 3B,C, where the high-level imagery group almost did not benefit from the instructions to imagine their body rotation as compared to sound condition. On the contrary, for the low-level kinesthetic imagery group such instructions increased their vection intensity scores when listening to the rotating bus sound. Similar pattern could be seen for AI and VI groups (Figures S1, S2).

**Figure 4:**
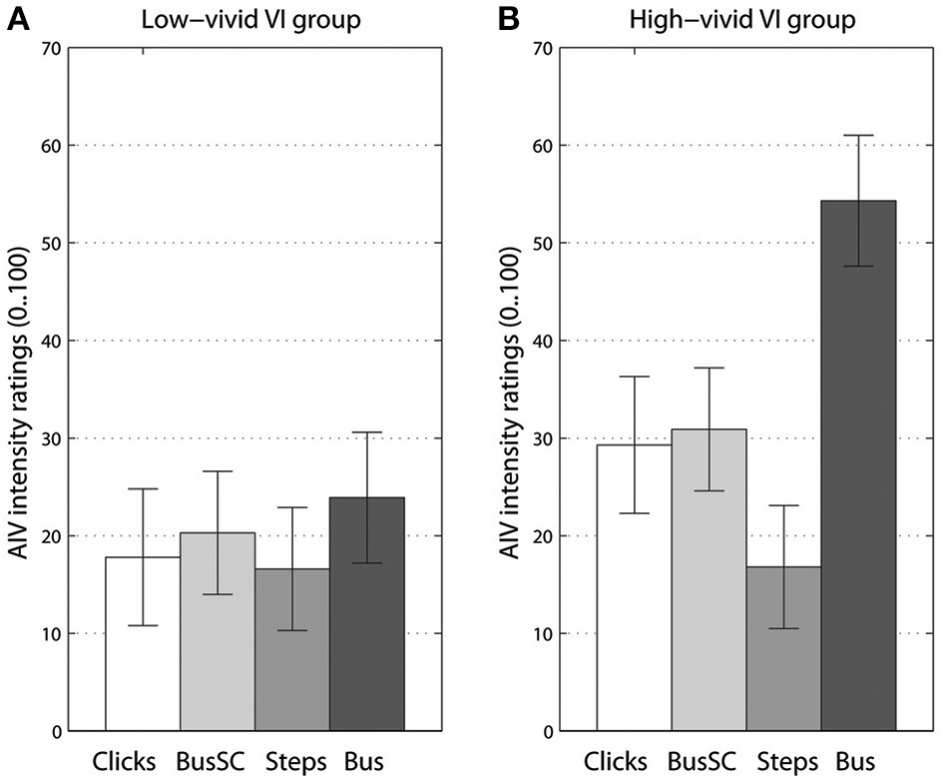
**Vection intensity ratings for different sound types separately for groups with low-vivid (A) and high-vivid (B) visual imagery (VI)**. Error bars represent standard error values.

For our first MANOVAs addressing acoustic landmark hypothesis, adding imagery vividness group as an additional between-subjects factor showed significant interaction with visual imagery [*F*_(6, 118)_ = 2.69, *p* < 0.017, Λ = 0.774, ${\hat{\eta }}_{P}^{2}$ = 0.12]. For univariate tests, this interaction with VI group was significant for AIV intensity ratings, [*F*_(2, 44)_ = 4.36, *p* < 0.016, ${\hat{\eta }}_{P}^{2}$ = 0.2]. These AIV intensity ratings split into high- and low-vivid visual imagery groups are shown in Figure 4. While in the low-vivid imagery group (panel **A**) AIV ratings almost did not differ between the types of the rotating sound, a more distinct picture emerged for the high-vivid imagery group (panel **B**). The smallest AIV ratings were given while being exposed to the footsteps sound, followed by the ambiguous stimuli (click train and the scrambled bus sound), and topping for the acoustic landmark type sound, the *“bus on idle.”* For auditory imagery, no multi- or univariate effects were observed (Figure S3). For kinesthetic imagery, the interaction was close to significance in univariate tests for AIV intensity ratings [*F*_(2, 40)_ = 2.5, *p* = 0.09, ${\hat{\eta }}_{P}^{2}$ = 0.1], showing similar pattern to high- and low-vivid VI groups (Figure S4).

**Figure 5:**
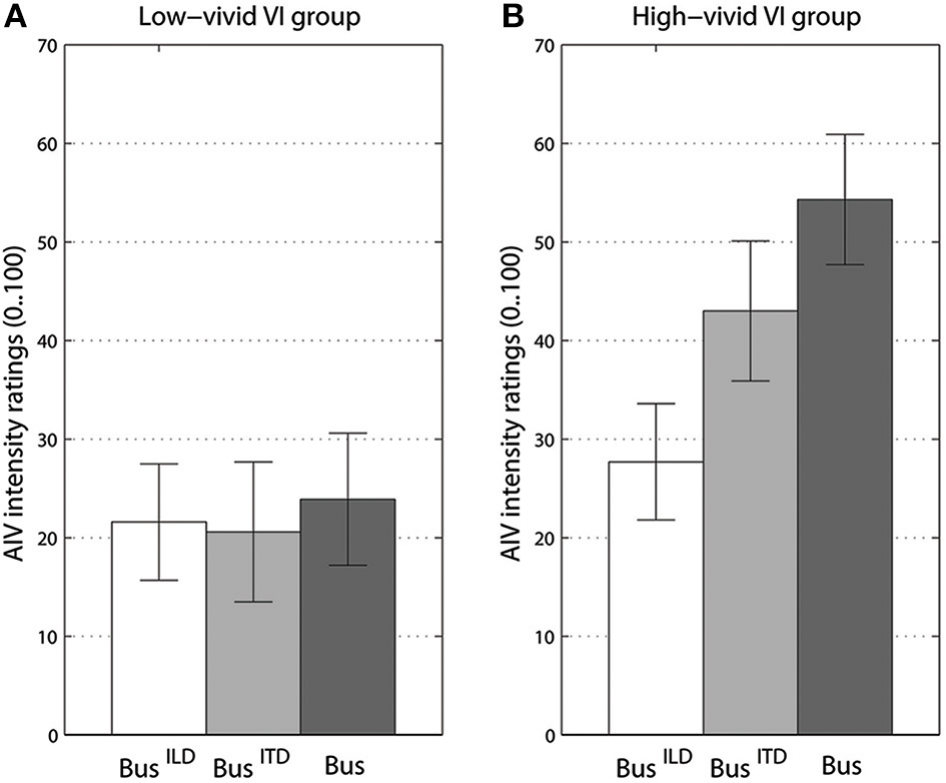
**Vection intensity ratings for different versions of a *“bus on idle”* separately for groups with low-vivid (A) and high-vivid (B) visual imagery (VI). Bus^ILD^** stands for high-pass filtered version, **Bus^ITD^** - or low-pass filtered version, **Bus** - for original. Error bars represent standard error values.

For our second MANOVA addressing frequency content of rotating stimuli, ratings from the vivid, and non-vivid imagery groups differed significantly only for visual imagery, [*F*_(4, 78)_ = 2.94, *p* < 0.05, Λ = 0.775, ${\hat{\eta }}_{P}^{2}$ = 0.13]. Specifically, strong interaction between imagery and frequency content was observed for AIV intensity ratings, [*F*_(2, 37)_ = 6.44, *p* < 0.005, ${\hat{\eta }}_{P}^{2}$ = 0.24]. The effect of frequency content factor also became stronger, [*F*_(2, 37)_ = 8.73, *p* < 0.001, ${\hat{\eta }}_{P}^{2}$ = 0.3]. The nature of interaction can be seen in Figure 5, where non-vivid imagery group ratings do not differ for any of three bus sound versions while the vivid imagery group show significant increase in vection as the acoustic cues become more prominent (see Figures S5, S6 for data on AI and KI groups). It should be noted that no such interaction could be observed for the ratings of sound object motion state. However, there was a trend (*p* = 0.08) showing that high-vivid imagery group rated bus sounds as more stationary, −2.8 (*SE* = 0.9) than non-vivid imagers −0.5 (*SE* = 0.9) (Figure S7).

## 4. Discussion

The present study addressed the contribution of perceptual and contextual cues to circular auditorily-induced vection. In line with our first hypothesis, the rotating sound stimuli identified by listeners as an auditory landmark, *“bus on idle,”* has led to significantly stronger levels of vection intensity. Probably due to loudspeaker rendering method, these results are even slightly higher (*M* = 39.1, *SE* = 5.7), than Larsson et al. (2004) data where listening to rotating stimuli containing a single acoustic landmark resulted in the mean AIV intensity of 36.3 as compared to 20.3 for a moveable sound [Larsson et al. (2004), Figure 12]. It should be noted that these levels are low in comparison with the acoustic fields containing two or three auditory landmarks, where the reported AIV intensity was at the levels of 40–50 on a 0–100 scale (Larsson et al., 2004; Väljamäe et al., 2009).

We also confirmed our second hypothesis showing that ITD cues are more instrumental than ILD for circular, yaw AIV. High-pass filtered version of the *“bus on idle”* sound (**Bus^ILD^**) has led to the significantly lower AIV intensity ratings than the unfiltered version (**Bus**), and it was comparable to the levels reported for artificial sounds (click train and scrambled version of the bus sound). Importantly, both **Bus^ITD^** and **Bus^ILD^** have been recognized by listeners as stationary sounds and did not significantly differ in ratings of sound object's motion. These results are in line with previous studies that showed interaction of vection experience and sound localization based on ITD but not on ILD cues (Väljamäe, 2009 and references therein). Otake et al. (2006) suggested that this susceptibility of ITD but not ILD discrimination in the presence of vection inducing stimuli may reflect different neural mechanisms or pathways involved in processing of these binaural cues. Interestingly, another study by this group used vertical optokinetic stimulation and found discrimination changes for both binaural cues but more prominent ones were again related to ITD tasks (Saito et al., 2006). However, it can be assumed that the integration of vestibular and auditory spatial information takes place at a stage of processing, where ITD and ILD cues had been integrated into a unified neural representation of auditory space, namely in the posterior parietal cortex, Lewald et al. (2002, 2004). While more focused investigation using filtered noise stimuli has to address neural mechanisms underlying such possible interaction between sound localization and AIV, one alternative explanation of these results can be related to the ecological acoustics perspective since low-frequency sounds typically correspond to bigger, and hence landmark-type objects. For example, Ohala (1996) describes such sound-symbolic relation, where high pitch was associated with “smallness” and non-threatening nature of an associated sound object and the reverse was found for the low pitch.

As predicted in our third hypothesis, the individual imagery vividness ability influenced the impact of different stimuli types on AIV. First, participants with low-vivid kinesthetic imagery showed clear benefit when the rotating bus sound was combined with the instructions to imagine self-rotation. In the case of the high-vivid kinesthetic imagery group, these instructions did not have such an effect. For visual or auditory imagery groups this imagery vividness effect was smaller suggesting that participants concentrated on kinesthetic nature of imagined self-rotation. Second, participants with high-vivid visual imagery made a prominent distinction between auditory landmark and moveable sound stimuli. Third, the observed effect of binaural cues contribution to AIV intensity was also pronounced only for the group with high-vivid visual imagery. No, or weaker, influence of kinesthetic imagery for these effects may demonstrate the top-down mechanism where auditory cues stimulate visual imagery of a rotating surround. It is different from other situations, like in Väljamäe et al. (2008a), where sound cues (a sound engine metaphor) directly stimulated kinesthetic imagery and where participants' KI vividness influenced the reported vection intensity. It should be also noted that auditory imagery group factor did not interact with any of the sound conditions. It is in line with the well-known “Perky effect” that shows the imagery perception interference with the real sensory input, in our case, with non-ambiguous acoustic cues. However, it is possible, that in visual or audio-visual experiments auditory imagery vividness may play a different role. For example, a rotating naturalistic visual scene containing stationary objects (landmarks) like a church or a fountain may trigger auditory imagery, especially if accompanying ambiguous sounds (white noise or ecological sounds near threshold) will be synchronously presented. To summarize, participants with a high-level of imagery vividness benefitted both from contextual (sound movability categorization) and perceptual (binaural) cues. At the same time, only the explicit instruction to imagine self-motion sensation had an affect on participants with low-vivid imagery.

Many studies addressed the effect of imagined self-motion vs. the actual one. It has been shown that in both cases similar neural mechanisms are employed, enabling the full class of motor imagery based Brain Computer Interface applications that rely on this fact (e.g., Wagner et al., 2013 and references therein). Importantly, vividness of self-rotation imagery has been shown to correlate with activation of the brain areas related to vestibular information processing Zu Eulenburg et al., 2013. A recent brain imaging study by Olivetti Belardinelli et al. (2009) addressed the issue of individual ability of imagery generation in different modalities. They found deactivation of modality-specific brain areas in low-vivid imagery participants as compared to activation of the same areas in high-vivid imagery group. The authors suggested that both groups demonstrated a different neural mechanism while involved in the imagery task. While high-vivid imagery group could be employing “unconscious simulations” of sensory activation, the low-vivid imagery group might be relying on “conscious semantic representations” that are based on the given verbal instructions. This line of thought well-explains the results in Väljamäe et al. (2008a), (Experiment 1) where low-vivid but not high-vivid kinesthetic imagery participants benefitted from the introduction of the “engine sound” metaphor when experiencing linear AIV.

In the line of thought of Olivetti Belardinelli et al. (2009), we may speculate that the facilitation of vection in high-vivid imagery group by external cues, either perceptual (binaural cues manipulation) or contextual (auditory landmarks), cannot be easily manipulated by instructions and it is rather automatic due to largely unconscious mechanisms. These mechanisms may be also related to the sensory conflict resolution that arises between stationary vestibular information and motion cues from other sensory modalities when simulating self-motion. Zu Eulenburg et al. (2013) reports that the task of imagined self-rotation and related reactivation of cortical vestibular areas is difficult as compared to other sensory modalities, since the “live” vestibular information may hinder recreation of imagined experience. In our case, vestibular imagery might be triggered in a more automatic way by real or imagery inputs provided by moving soundscapes or related visual imagery. Indeed, the instructions to imagine self-rotation did not alter significantly already present experience of AIV in high-vivid imagery group. At the same time, the low-vivid imagery group benefitted from instructions and were assisted by external cues due to a more conscious strategy. Alternatively, the observed instruction effect in the low-vivid imagery group might be also related to attention mechanisms. For example, Ikeda et al. (2012) showed that focusing attention on auditory or visual cues related to motor imagery task (finger movement) enhanced excitability of the primary motor cortex.

Although not studied extensively, the idiosyncratic nature of vection responses has been addressed in the past. Kennedy et al. (1996) showed that some participants exhibited greater sensitivity to vection inducing stimuli than others, and this may be related to the individual differences in the vestibular thresholds (Lepecq et al., 1999). Participants can also differ in the weight or attention they are giving to different sensory modalities. An unpublished pilot study in Väljamäe (2007) used the field dependence/independence (FD/FI) measure to determine sensory modality preference. The results showed that non-visual cues might be more vection inducing for FI than for FD participants. Dedicated studies using auditory cues in combination with visual and vestibular cues for inducing vection may further advance the understanding of different mechanisms involved in forming the multisensory sensation of vection and the related individual differences.

Even though vection sensation can be produced purely by moving acoustic fields, the specificity of such easily breakable auditorily illusion makes it interesting only for a limited set of specific applications such as auditory vision substitution systems (Väljamäe and Kleiner, 2006), interactive audio-books (Röber et al., 2006), audio only games (Röber and Masuch, 2005) or multimedia based mobile dramas (Hansen et al., 2012). However, the use of sound in combination with visual and vestibular cues significantly increases vection, especially when visual cues are weak. For example, sound specific benefits include its dominance in conveying the dynamics of a multisensory scene and the applications where visual cues are reduced or not available to the user (Väljamäe et al., 2008b). Providing both spatial and temporal sensory cues, sound can also carry contextual, high-level information enhancing the vividness of illusory self-motion, such as an engine sound metaphor serving as implied motion cue (Väljamäe et al., 2008a). To conclude, sound cues for AIV are an important but often overlooked component in the design of motion simulation and gaming applications based on synthetic multisensory environments.

## Author contributions

Aleksander Väljamäe (AV) and Sara Sell (SS) designed the experiment; Sara Sell collected data; Aleksander Väljamäe analyzed data; Aleksander Väljamäe and Sara Sell discussed the results and wrote the manuscript.

## Funding

The last author of this paper received funding from Marie Curie Actions of the European Unions Seventh Framework Programme (FP7/2007-2013) under REA GA-303172.

### Conflict of interest statement

The authors declare that the research was conducted in the absence of any commercial or financial relationships that could be construed as a potential conflict of interest.
