# Supplementary material for: The influence of imagery vividness on cognitive and perceptual cues in circular auditorily-induced vection
Source: Front Psychol. 2014 Dec 3;5:1362. doi: 10.3389/fpsyg.2014.01362 (PMC4253967; doi:10.3389/fpsyg.2014.01362)
Supplement: Supplementary file 1 [file Presentation1.PDF]

Frontiers in Psychology

Research Article
13 October 2014

Supplementary Material: The influence of imagery vividness on cognitive and perceptual cues in circular auditorily-induced vection

Aleksander Väljamäe [1,*], Sara Sell [2,3,4]

[1] *Decision, Emotion and Perception lab, Department of Behavioural Sciences and Learning, Linköping University, Campus Valla, 581 83, Linköping, Sweden*
[2] *National Center for Rehabilitative Auditory Research, Portland, Oregon, USA*
[3] *Pacific University, Forest Grove, Oregon, USA*
[4] *James Madison University, Harrisonburg, Virginia, USA*

Correspondence*:
corresponding Author
Decision, Emotion and Perception lab, Department of Behavioural Sciences and Learning, Linköping University, Campus Valla, 581 83, Linköping, Sweden,
aleksander.valjamae@liu.se

1 SUPPLEMENTARY TABLES AND FIGURES

1.1 THE IMAGINED BODY ROTATION EFFECT - AUDITORY AND VISUAL IMAGERY GROUPS

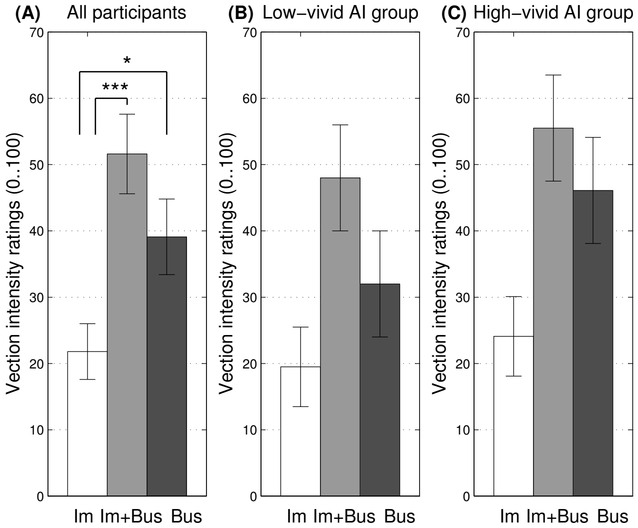

Figure S1. The effect of imagined body rotation with or without auditory stimuli (**A**), and data split for low-vivid (**B**) and high-vivid (**C**) auditory imagers (AI). **Im** stands for imagery of self-rotation, **Bus** - for original *"bus on idle"* sound, and **Im+Bus** for imagery combined with the rotating bus sound. Significant differences from Bonferroni-corrected pairwise comparisons are marked at $p < 0.05$ (*), and at $p < 0.005$ (***) levels. Error bars represent standard error values.

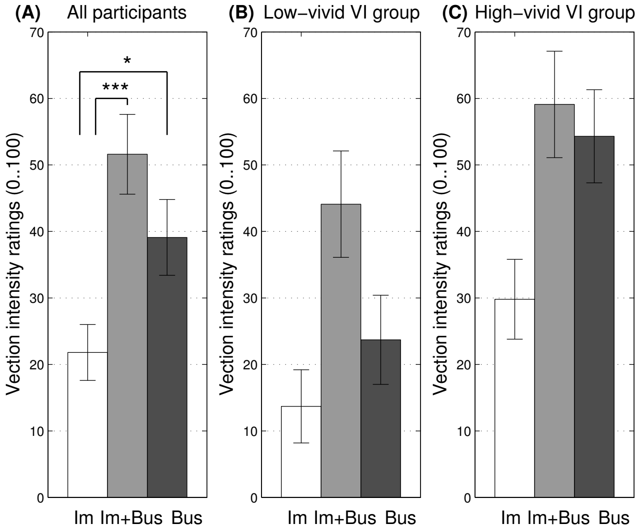

Figure S2. The effect of imagined body rotation with or without auditory stimuli (**A**), and data split for low-vivid (**B**) and high-vivid (**C**) visual imagers (VI). **Im** stands for imagery of self-rotation, **Bus** - for original *"bus on idle"* sound, and **Im+Bus** for imagery combined with the rotating bus sound. Significant differences from Bonferroni-corrected pairwise comparisons are marked at $p < 0.05$ (*), and at $p < 0.005$ (***) levels. Error bars represent standard error values.

1.2 THE SOUND TYPE EFFECT - AUDITORY AND KINESTHETIC IMAGERY GROUPS

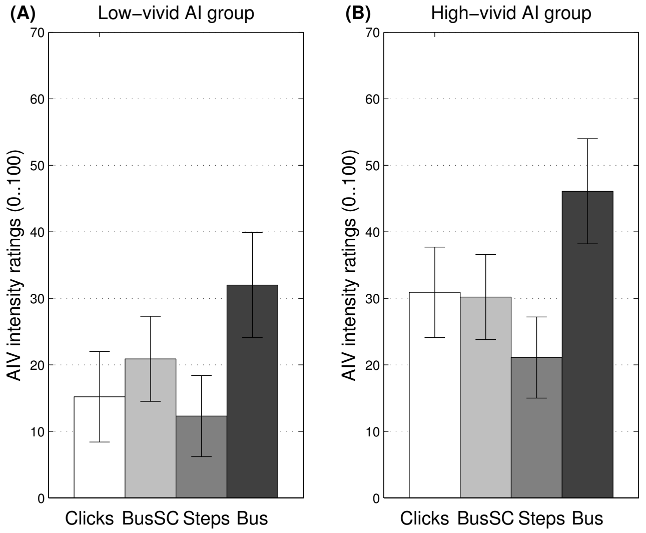

Figure S3. Vection intensity ratings for different sound types separately for groups with low-vivid (**A**) and high-vivid (**B**) auditory imagery (AI) . Error bars represent standard error values.

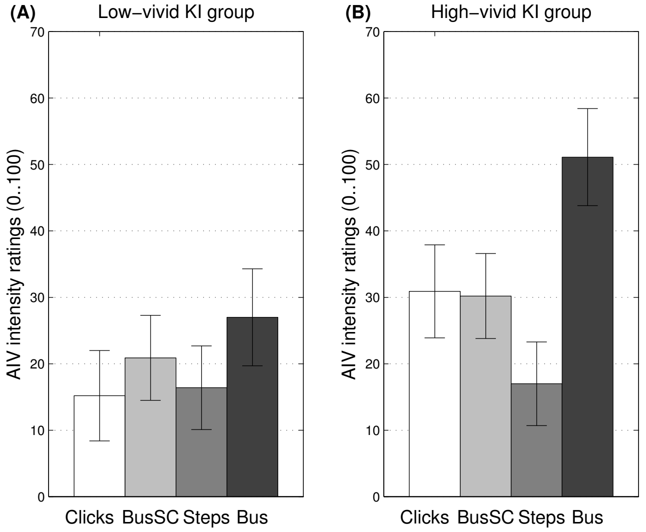

Figure S4. Vection intensity ratings for different sound types separately for groups with low-vivid (**A**) and high-vivid (**B**) kinesthetic imagery (KI) . Error bars represent standard error values.

1.3 THE BINAURAL CUES EFFECT - AUDITORY AND KINESTHETIC IMAGERY GROUPS

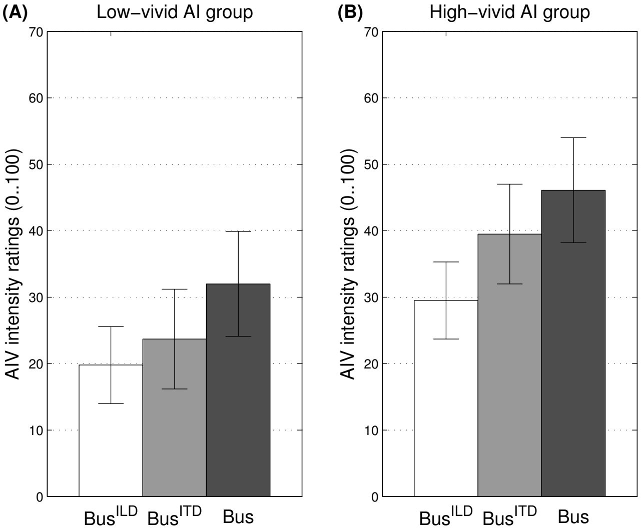

Figure S5. Vection intensity ratings for different versions of a *"bus on idle"* separately for groups with low-vivid (**A**) and high-vivid (**B**) auditory imagery (AI). **Bus**$^{\mathbf{ILD}}$ stands for high-pass filtered version, **Bus**$^{\mathbf{ITD}}$ - or high-pass filtered version, **Bus** - for original. Error bars represent standard error values.

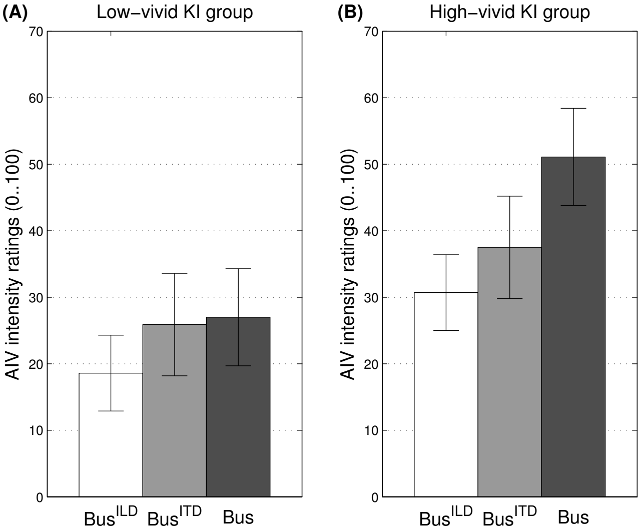

Figure S6. Vection intensity ratings for different versions of a *"bus on idle"* separately for groups with low-vivid (**A**) and high-vivid (**B**) kinesthetic imagery (KI). **Bus**$^{\mathbf{ILD}}$ stands for high-pass filtered version, **Bus**$^{\mathbf{ITD}}$ - or high-pass filtered version, **Bus** - for original. Error bars represent standard error values.

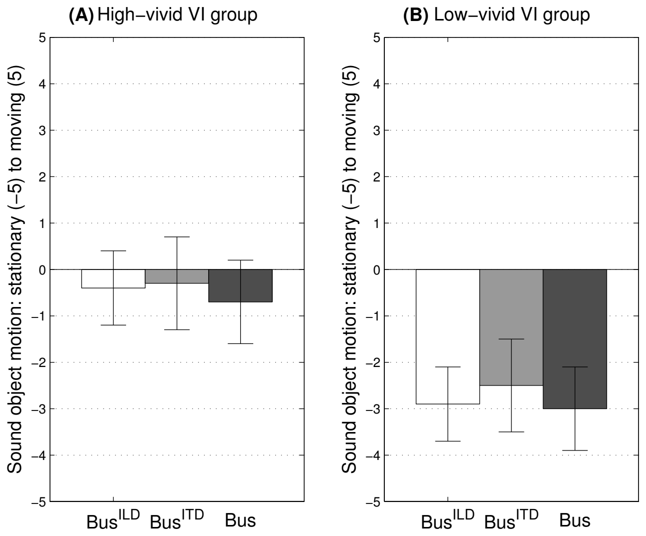

Figure S7. Sound object's perceived motion ratings for different versions of a *"bus on idle"* separately for groups with low-vivid (**A**) and high-vivid (**B**) visual imagery (VI). **Bus$^{\mathbf{ILD}}$** stands for high-pass filtered version, **Bus$^{\mathbf{ITD}}$** - or high-pass filtered version, **Bus** - for original. Error bars represent standard error values.

1.4 FIGURES IN THE MAIN MANUSCRIPT

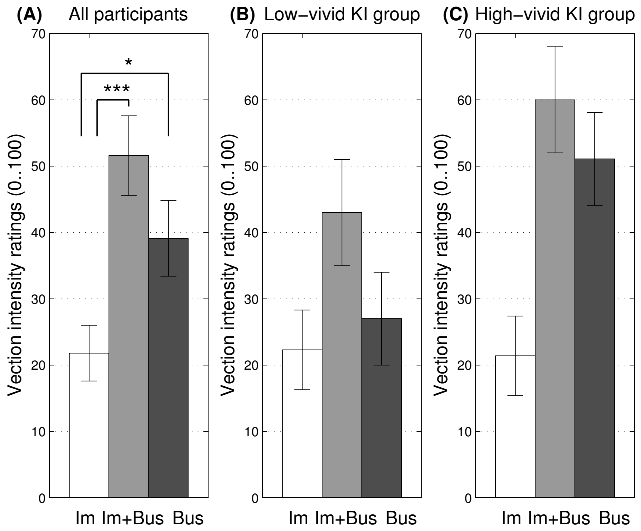

Figure 3. The effect of imagined body rotation with or without auditory stimuli (**A**), and data split for low-vivid (**B**) and high-vivid (**C**) kinesthetic imagers. **Im** stands for imagery of self-rotation, **Bus** - for original *"bus on idle"* sound, and **Im+Bus** for imagery combined with the rotating bus sound. Significant differences from Bonferroni-corrected pairwise comparisons are marked at $p < 0.05(*)$, and at $p < 0.001(***)$ levels. Error bars represent standard error values.

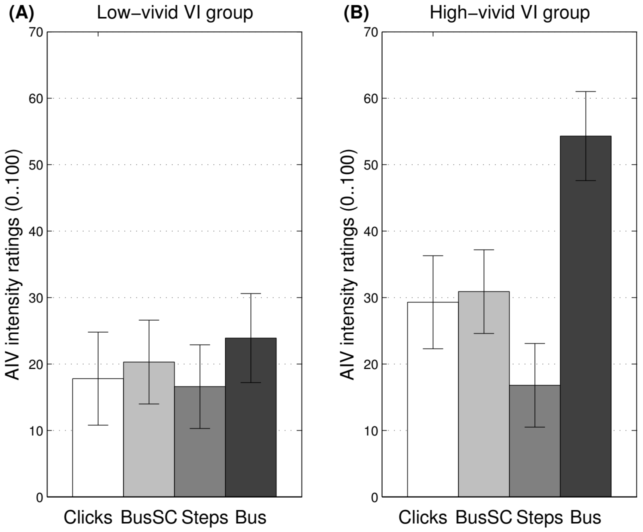

Figure 4. Vection intensity ratings for different sound types separately for groups with low-vivid (**A**) and high-vivid visual imagery (**B**). Error bars represent standard error values.

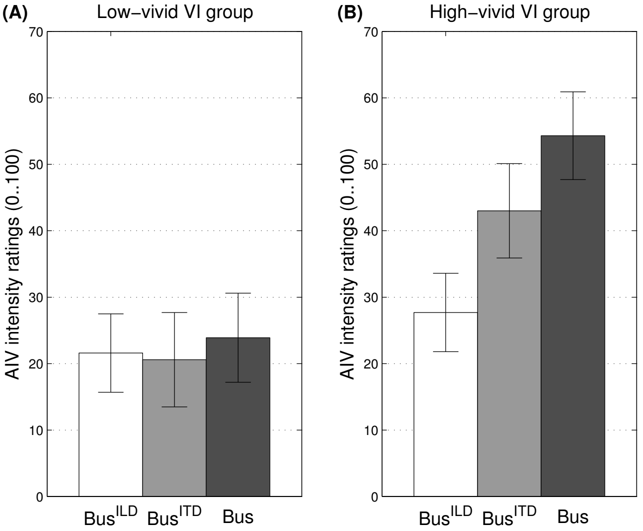

Figure 5. Vection intensity ratings for different versions of a *"bus on idle"* separately for groups with low-vivid (**A**) and high-vivid visual imagery (**B**). **Bus**$^{\mathbf{ILD}}$ stands for high-pass filtered version, **Bus**$^{\mathbf{ITD}}$ - or high-pass filtered version, **Bus** - for original. Error bars represent standard error values.
